# Supplementary material for: Amber Extract Reduces Lipid Content in Mature 3T3-L1 Adipocytes by Activating the Lipolysis Pathway
Source: Molecules. 2021 Jul 30;26(15):4630. doi: 10.3390/molecules26154630 (PMC8348738; doi:10.3390/molecules26154630)
Supplement: Supplementary file 1 [file molecules-26-04630-s001.zip › molecules-1288501-supplementary.pdf]

Supplementary Materials

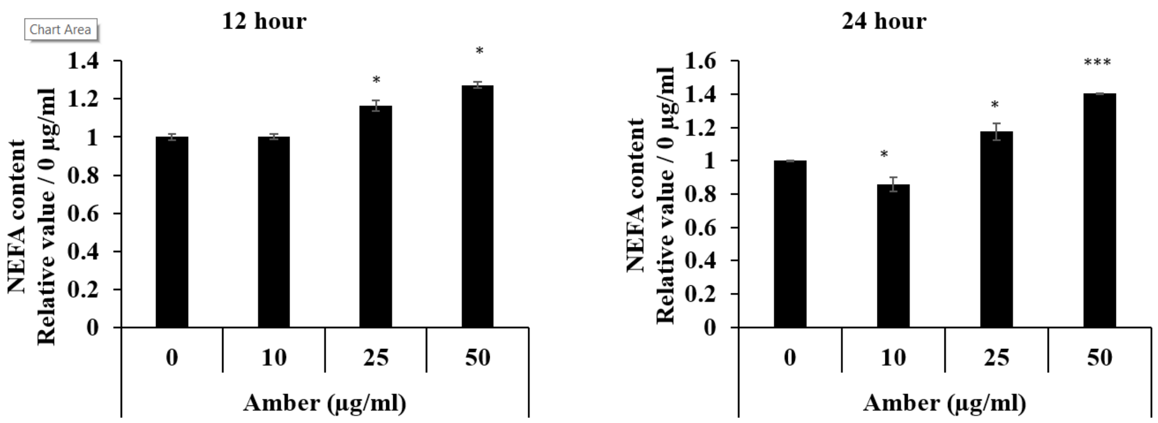

Figure S1 Amber increased the NEFA content in medium after 12- and 24-hour treatment.

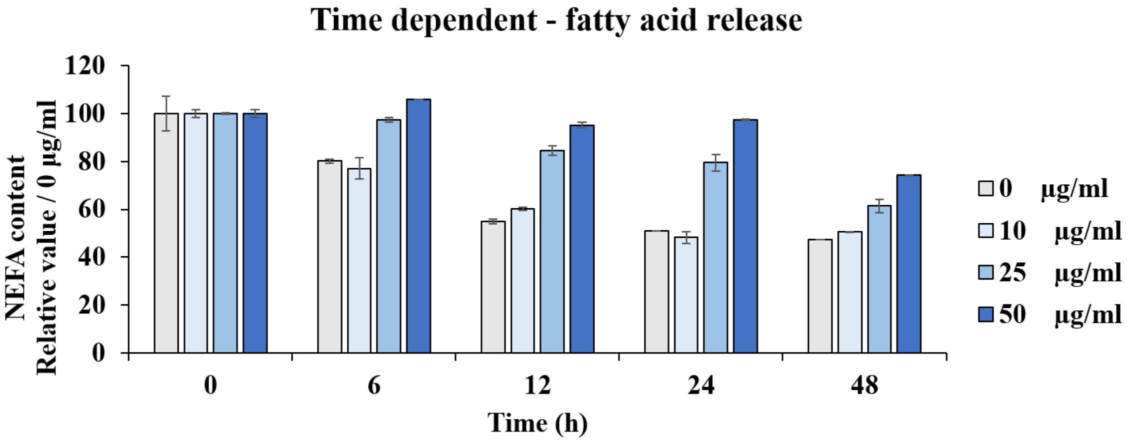

Figure S2 Amber dose-dependently slow down the decrease of NEFA in medium.
